# A Sample-to-Sequence Protocol for Genus Targeted Transcriptomic Profiling: Application to Marine *Synechococcus*

**DOI:** 10.3389/fmicb.2016.01592

**Published:** 2016-10-14

**Authors:** Frances D. Pitt, Andrew Millard, Martin Ostrowski, Suat Dervish, Sophie Mazard, Ian T. Paulsen, Mikhail V. Zubkov, David J. Scanlan

**Affiliations:** ^1^School of Life Sciences, University of WarwickCoventry, UK; ^2^Warwick Medical School, University of WarwickCoventry, UK; ^3^Department of Biochemistry and Biomolecular Science, Faculty of Science and Engineering, Macquarie UniversitySydney, NSW, Australia; ^4^Sydney Cytometry, Centenary Institute, University of SydneySydney, NSW, Australia; ^5^National Oceanography CentreSouthampton, UK

**Keywords:** marine microbiology, *Synechococcus*, flow cytometry, transcriptomics, fluorescence activated cell sorting, RNA

## Abstract

Recent studies using whole community metagenomic and metatranscriptomic approaches are revealing important new insights into the functional potential and activity of natural marine microbial communities. Here, we complement these approaches by describing a complete ocean sample-to-sequence protocol, specifically designed to target a single bacterial genus for purposes of both DNA and RNA profiling using fluorescence activated cell sorting (FACS). The importance of defining and understanding the effects of a sampling protocol are critical if we are to gain meaningful data from environmental surveys. Rigorous pipeline trials with a cultured isolate, *Synechococcus* sp. BL107 demonstrate that water filtration has a well-defined but limited impact on the transcriptomic profile of this organism, whilst sample storage and multiple rounds of cell sorting have almost no effect on the resulting RNA sequence profile. Attractively, the means to replicate the sampling strategy is within the budget and expertise of most researchers.

## Introduction

Next generation sequencing (NGS) techniques offer huge potential for the genetic exploration of marine bacteria. Initial Sanger-based genome sequencing of marine isolates (Palenik et al., 2003; Rocap et al., 2003; Giovannoni et al., 2005) has been superseded by NGS approaches capable of sequencing multiple genomes simultaneously (Kashtan et al., 2014; Billerbeck et al., 2016). Corresponding marker gene studies have revealed the spatial partitioning and temporal succession of marine bacterial communities (Fuhrman and Steele, 2008; Gilbert et al., 2012; Teeling et al., 2012; Needham and Fuhrman, 2016), highlighting the diversity and geographic distribution of specific marine microbes (Johnson et al., 2006; Zwirglmaier et al., 2008; Gómez-Pereira et al., 2010; Farrant et al., 2016), exposing the extent and dynamics of the rare microbial biosphere (Campbell et al., 2011; Welch and Huse, 2011). Advances in cell sorting and single cell sequencing circumvent the limitations of culturing to study the genomes of keystone marine species (Zehr et al., 2008; Tripp et al., 2010; Heywood et al., 2011; Worden et al., 2012; Kashtan et al., 2014; Mende et al., 2016) while the greater sequencing depth achieved by the latest NGS platforms unlocks the potential for detailed metagenomic and metatranscriptomic studies of natural microbial communities (Cuvelier et al., 2010; Shi et al., 2010; Batmalle et al., 2014; Satinsky et al., 2014; Aylward et al., 2015; Shilova et al., 2016). Together with large-scale ocean sampling surveys (Nicholls, 2007; Hurwitz and Sullivan, 2013; Armbrust and Palumbi, 2015; Rees et al., 2015; Sunagawa et al., 2015) and well-established ocean time stations (Phillips and Joyce, 2007; Karl and Church, 2014) the means to tap the oceans entire genomic potential is seemingly within our grasp.

Environmental sampling approaches for both metagenomic and metatranscriptomic studies are clearly shaped by the questions researchers wish to ask yet constrained by an understanding of current limits in technology. The task of assessing the genomic potential of the marine picoplankton community has led to much of the early pioneering work going into the development and validation of sampling strategies and methodologies. In all cases, a specific size fraction from the water sample is filtered onto a flat membrane or pleated filter, which is preserved for DNA extraction and sequenced at a later date. The introduction of bias into the profile can be observed with the choice of cell size fractionation (Craig, 1986; Padilla et al., 2015), the method in which the cells are lysed and the manner in which material is amplified–if yield requirements are not met for sequencing (Batmalle et al., 2014). This entire process becomes even more complex when we consider using the same material for combined DNA and RNA studies. Since the half-life stability of bacterial mRNA is on the order of minutes (Steglich et al., 2010) the amount of time taken to collect and concentrate biomass from the environment is a critical factor. However, reducing the time for collection and concentration limits the total yield of biomass, with the consequence that the obtained material must then be amplified to meet DNA/RNA library preparation requirements. Important considerations need to be made, not only in the time and manner in which the material is collected, filtered and preserved but also the handling of the RNA post extraction—all of which have the potential to elicit a transcriptional response and impact on the recovered RNA profile. Considerable effort in recent years has gone into the development of environmental transcriptomic methodologies, with a view to understanding how the sampling process impacts the data produced (Stewart et al., 2010; Ottesen et al., 2011).

While large-scale marine metagenomics projects are beginning to reveal the genomic potential of distinct communities, there are still major hurdles to overcome in understanding functional adaptation. Transcriptomics provides the means to address two of the current major challenges in marine microbiology: (i) to develop an understanding of how the genomic blueprint of an organism is dynamically expressed under a set of environmental conditions, and (ii) to begin to uncover the role of the plethora of hypothetical genes i.e., those with no known function, present in all microbial genomes/metagenomes. Correlating gene expression levels with changing environmental conditions provides a means to group known and unknown genes into functional regulons. However, the ability to assign functional traits to a specific genus or group within the community is underpinned by an ability to map reads accurately to the correct taxonomic group. Community-wide analyses of sequence data may provide an overview of biological functions in organisms that dominate DNA but fail to assign genes and associated traits to keystone species involved in critical environmental processes, such as primary production and nitrogen fixation (Jardillier et al., 2010). This is of particular importance when attempting to dissect the functional potential of genes from marine microbes and to link molecular adaptations with the eco-physiological limits of species and ecotypes.

Genome sequencing of both laboratory isolates and single cells has built a mosaic picture of core genes common to many, and auxiliary genes specific to a few. The auxiliary gene sets present some of the most interesting, perhaps niche-defining features of the genomes. However, their presence on mobile genomic islands makes the taxonomic assignment of reads for some genes potentially problematic since their phylogenetic origins may lie in an entirely different genus (Coleman et al., 2006; Dufresne et al., 2008; Penn et al., 2009). With this in mind, the selective isolation of a single genus from an environmental sample, made possible by fluorescence activated cell sorting (FACS), could lead to a more accurate assignment of transcriptomic reads. Targeting a specific planktonic group via FACS for metagenomic sequencing has already been shown to improve specific gene coverage, particularly important if the group under study is a minority player within the whole community (Cuvelier et al., 2010; Thompson et al., 2013; Batmalle et al., 2014). However, there remain significant challenges in both RNA recovery and amplification bias before FACS targeted transcriptomics can be realized. To address these challenges, we conducted a series of experiments designed to validate a complete ocean-to-sequence protocol with a proof of concept application on marine *Synechococcus*.

Cyanobacteria of the genus *Synechococcus* are a ubiquitous component of the marine photosynthetic community, significantly contributing to primary productivity (Jardillier et al., 2010; Flombaum et al., 2013). Whilst numerically less abundant than their sister taxon *Prochlorococcus*, they have a wider geographic distribution occupying diverse marine habitats from large mid-ocean oligotrophic gyres to nutrient-enriched coastal waters, upwelling systems and polar regions (Li et al., 1983; Partensky et al., 1999; Saito et al., 2005; Cottrell and Kirchman, 2009). Through genome sequencing, phylogenetic marker amplicon analysis and metagenomic recruitment studies, a clear picture of the genus is emerging where the existence of multiple ecotypes inhabiting distinct ocean niches prevails (Dufresne et al., 2008; Zwirglmaier et al., 2008; Farrant et al., 2016; Sohm et al., 2016). Factors controlling *Synechococcus* abundance and activity have been explored in the form of *in situ* rate measurements and candidate marker genes representing key processes (Fuller et al., 2005; Lindell et al., 2005; Grob et al., 2015). The obvious limitation of this approach is that it draws on known expression analyses of cultured representatives and relies on the assumption that cells perform in a similar manner in the environment. A targeted *in situ* transcriptomic approach would ultimately aim to identify clade-specific gene sets under dynamic expression, to interpret what genomic factors are most important in controlling both geographical and temporal distribution patterns and contributing to their activity in global nutrient cycles.

The key challenges for a sampling protocol are to process sufficient sample volume over a short timeframe to yield enough material for amplification-free sequencing without significantly altering the *in situ* transcriptional profile of the target cells. In the nutrient deplete oligotrophic gyres, *Synechococcus* cell numbers can fall to as low as 1 × 10^3^ cells ml^−1^ (Partensky et al., 1999; Zubkov et al., 2007; Zwirglmaier et al., 2008) which can represent < 1% of the cells in the entire community. Whereas, 1–20 l of seawater would provide adequate material for a whole microbial community-level RNA extraction, far larger (>10-fold), volumes of water would be required to yield sufficient flow sorted *Synechococcus* cells for the extraction of both DNA and RNA. In addition, the combined effects of both concentration and cell sorting on a transcription profile are poorly described and need to be considered carefully in order to draw meaningful biological information from expression data.

To address these issues we designed a new high-speed filtration and cell concentration apparatus using accessible and relatively low-cost components and validated the protocol for transcript recovery from a simulated open ocean filtration and sorting experiment against that of a control using a single reference strain of *Synechococcus* (BL107). The transcription profile of BL107 was examined at each stage of the protocol and compared with a control profile where no processing had occurred. We were able to demonstrate that (i) all major changes to the transcript profile occur within the filtration phase and not the cell sorting phase (ii) changes to the transcript profile are limited and well-defined, and (iii) rapid cell filtration can be achieved via a low cost, easily maintained filtration rig that does not require specialist operation.

## Materials and methods

### High speed water filtration (SWiFT) apparatus design and operation

**Figure 1:**
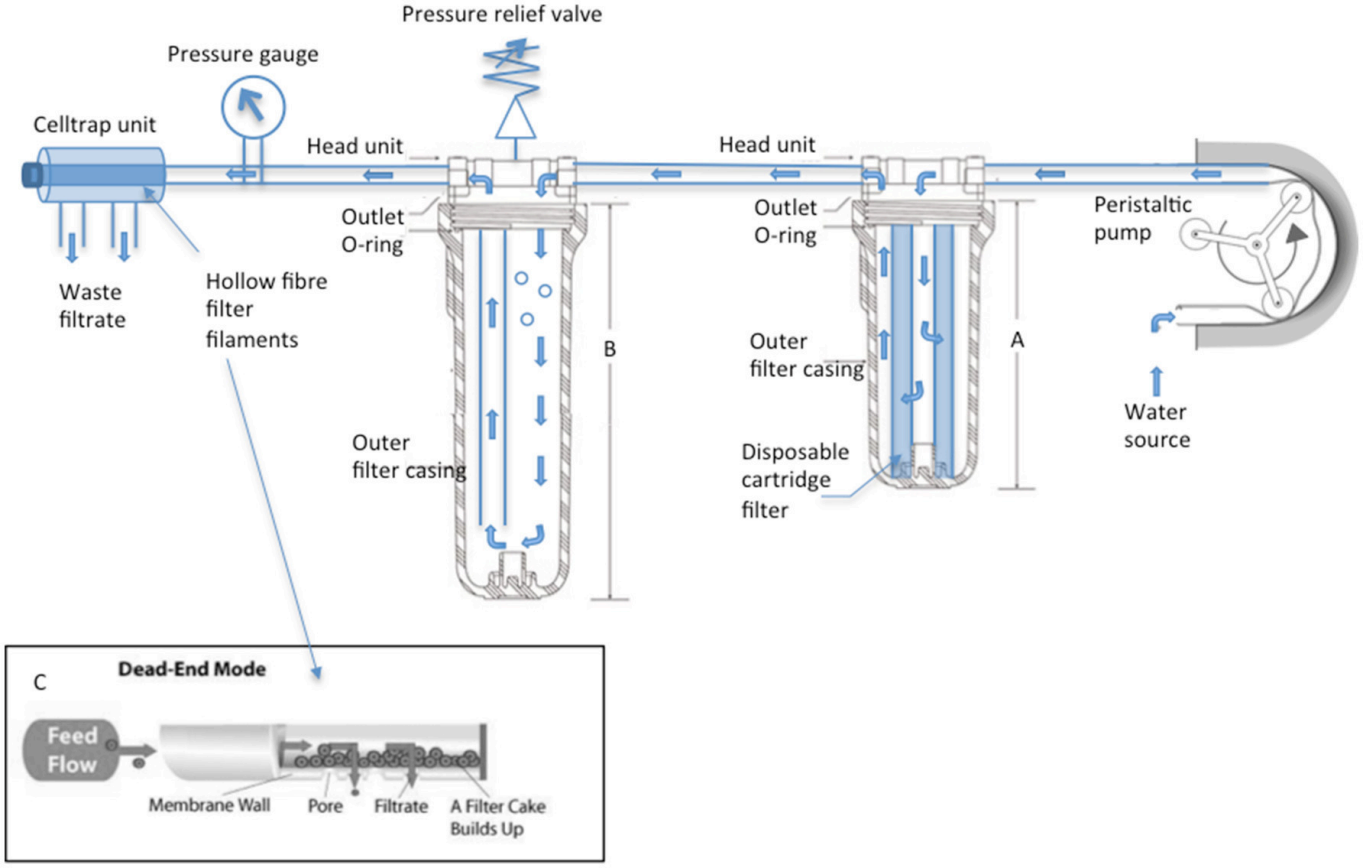
**SWiFT cell capture rig technical specifications**. A Watson Marlow 620SN/R encased pump draws source water through the apparatus, water flow indicated by blue arrows. Clear water filtration unit **(A)** 10″ houses the disposable supaplete II 35 micron pre-filter (Amazon filters) while **(B)** 20″ acts as a bubble trap once air is purged from the chamber via the adjustable valve. High volume Celltrap units connect to the main body via luerlok connection to perform a dead-end filtration. **(C)** Cells are recovered from the disconnected CT by drawing filtrate back through the filaments with a 50 ml luer lock syringe (VWR).

The SWiFT cell capture rig (Figure 1) is designed to take seawater directly from source. Seawater undergoes a two-phase filtration: first passing through a 35 μm cartridge filter housed within a standard consumer grade under-counter water filter unit before passing through a Memteq High Volume Celltrap (HVCT) 0.2 μm filter unit. Filter units are connected via silicone tubing attached with jubilee clips to the barb connectors. Water is driven through the apparatus via a Watson Marlow 620SN/R cased peristaltic pump. An in-line pressure gage allows the user to monitor pressure increase as cells build up within the HVCT, avoiding HVCT overloading. The SWiFT rig is adaptable for use with alternative peristaltic pump models, subject to their liters/min water pumping capacity. HVCT units are pre-packed sterile. Following a single round of filtration and cell recovery the unit is disposed and not re-used. Before entering the HVCT water enters a large transparent housing unit which, once filled with flow through water, acts as a bubble trap drawing water from the bottom while inflow occurs at the top. Avoidance of air entering the HVCT is a critical step as once a bacterial film begins to line the HVCT filaments, bubbles are unable to pass through with the resulting accumulation of bubbles blocking flow through the HVCT over time.

### *Synechococcus* sp. BL107 sampling

A mid-exponential phase culture of *Synechococcus* sp. BL107 grown in artificial seawater medium (ASW; Wyman et al., 1985) at 22°C with 10 μmol photons m^−2^ s^−1^ continuous light was divided in two. Half of the culture was centrifuged at 4754 × g for 4 min, and three RNA extractions performed immediately. The remainder of the culture was diluted in ASW to represent a concentration of *Synechococcus* more akin to those found in natural environments of the North Atlantic (ca. 3 × 10^4^ cells ml^−1^; Zubkov et al., 1998) and filtered using the SWiFT rig (Figure 1) for 15 min. Cells were recovered in suspension from the HVCT unit by drawing back through the trap 10 ml filtrate with a syringe followed immediately by flash freezing in liquid nitrogen. Total filtration time inclusive of water transfer to rig and cell freezing amounted to 20 min. Frozen samples were stored at −80°C for 16 weeks to replicate typical on-ship sample transit times following the Atlantic Meridional Transect ocean sampling program.

### Flow cytometric sorting of *Synechococcus* sp. BL107

**Figure 2:**
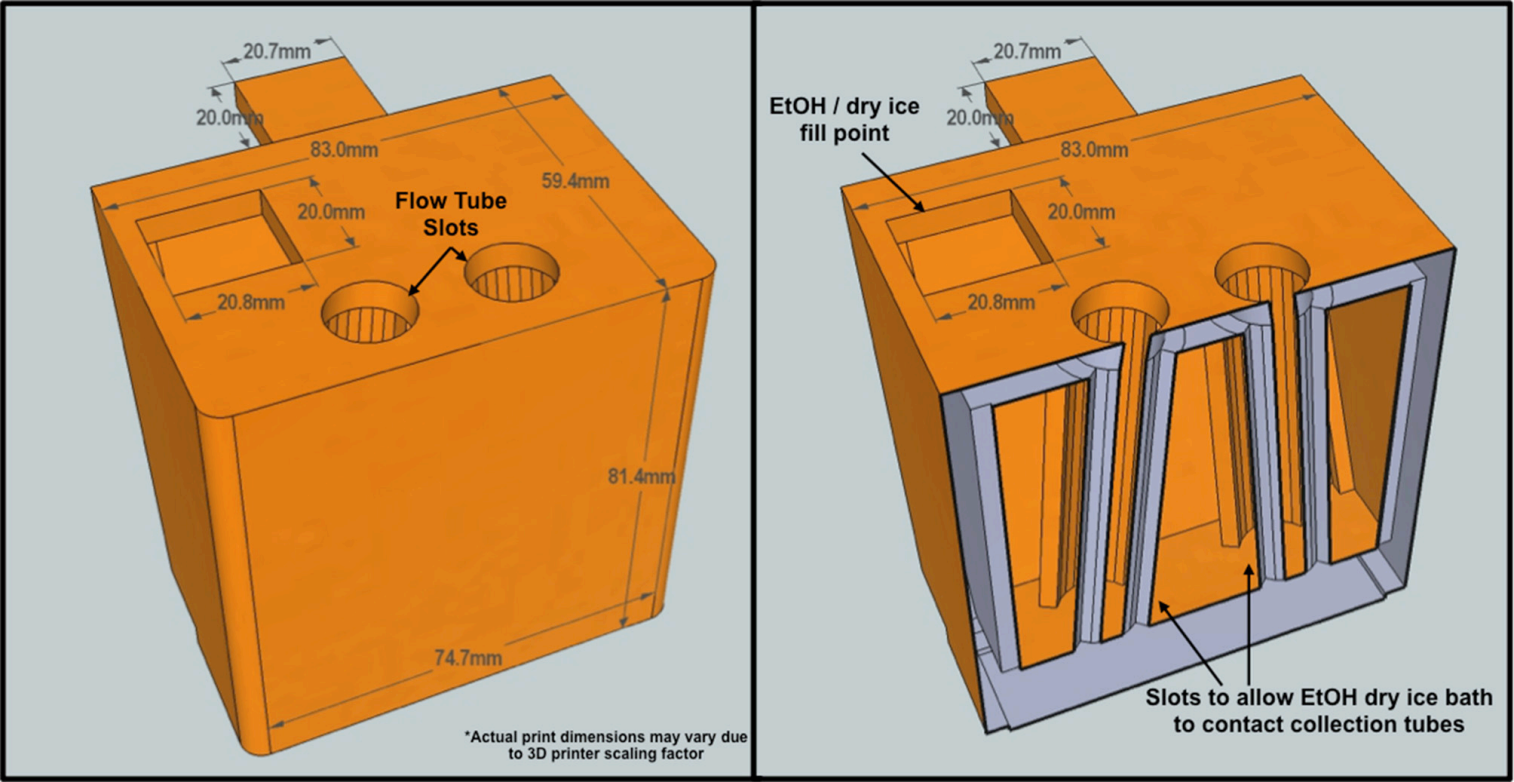
**Custom BD Influx flow tube holder cross-section**. The tube holder was designed to support a two-way sort program. Flow tubes are suspended within a hollow chamber designed to bring them into direct contact with a dry ice ethanol bath. Sorted cells freeze on contact with inserted flow tubes.

*Synechococcus* sp. BL107 cells were recovered from the frozen filtrate by FACS using a BD Influx™ Cell Sorter (BD Biosciences). Before each round of sorting both sheath line and sample line were cleaned with two rounds of FACSclean and FACSrinse each taking 30 min to ensure purity of both the sample line and sheath fluid. Filter sterilized (0.22 μm) ASW without phosphate/nitrate, and trace metals was used as sheath fluid. A 70 μm nozzle was used in combination with a fluidic pressure of 65 psi during sorting. For the single round of sorting, 0.5 ml of frozen filtrate was defrosted on ice for 10 min in the presence of potassium citrate (30 mM final concentration) and SYBRGold nucleic acid stain, final concentration 10^−2^ concentration of the commercial stock (Invitrogen; Marie et al., 1997). Cells were excited using a 488 nm, 200 mW laser with a standard filter setup. The BD Influx™ was set to one-drop pure sort mode (highest sort purity for this instrument) and *Synechococcus* sp. BL107 cells gated and separated based on their green (530 nm) and orange (580 nm) fluorescence. Cells leaving the sort stream were diverted into a custom collection chamber designed to support a Falcon 5 ml polypropylene round bottom flow tube within a bath of dry ice and ethanol (Figure 2). Cells were instantly frozen on contact with the collection tube preventing prolonged thaw times. Each thawed and stained aliquot of cells took 10 min for sort completion. A total of 2.5 × 10^7^ cells were sorted for each of the three single sort samples. For the two consecutive rounds of cell sorting, the first round served as a basic enrichment. Cells were not stained, with sort gating and selection solely based on their phycoerythrin auto-fluorescence (580 nm). In the second round of flow sorting cells were stained and sorted as described above. A total of 1.5 × 10^7^ cells were flow-sorted for the two double sort samples. Sort purity was regularly assessed as described by Zubkov et al. (2007). Sorted material was 99% enriched with recovery ranging from 85 to 95%.

### Flow cytometric counts of *Synechococcus* sp. BL107

*Synechococcus* sp. BL107 cell counts for the purpose of pre-filter retention testing were performed on a FACScan flow cytometer (BD Biosciences). Cells were excited using a 488 nm 20 mW laser with detection based on 580 nm auto-fluorescence properties (Olson et al., 1993). To calculate cells ml^−1^, the material recovered through HVCT was diluted 1000-fold in ASW medium spiked with a known concentration of fluorescent beads (Sphero Nile Red Beads 1.7–2.2 μm). From the bead counts the total number of beads detected in a fixed amount of time was calculated as a percentage of the total known bead concentration. Cell concentration could then be calculated based on the assumption that the same percentage of cells pass through the laser compared to beads.

### RNA extraction and next generation sequencing

RNA extraction was based on a variation of the protocol described in Gilbert et al. (2000). Briefly, frozen sorted cells were filtered onto a 13 mm 0.2 μm Supor® filter (Pall) and re-suspended in 0.4 ml solution A (0.3 M sucrose, 0.01 M sodium acetate), 0.4 ml solution B (0.01 M sodium acetate pH 4.2 2% SDS), 0.8 ml acidic phenol (pH 4.3) then bead beaten in Lysing Matrix E tubes (MP Biomedicals) for 45 s, transferred to ice for 45 s and repeated. The aqueous phase was then purified using the RNA Clean & Concentrator™ −5 kit (Zymo Research, CA) with a final elution volume of 15 μl. Samples were then treated with Turbo DNase (Ambion) for 30 min followed by a second round of purification with the RNA Clean & Concentrator™ −5 kit. Total RNA quality and yield were assessed using Bioanalyzer RNA 6000 nano and pico chips (Agilent) and QuantiFluor RNA System (Promega) analysis, respectively. Total RNA was prepared for sequencing using the ScriptSeq v2 (Epicentre) library workflow for the HiSeq2000 2 × 100 bp reads (V3 chemistry). Samples were sequenced to various depths by the NERC Biomolecular Analysis Facility (NBAF) located at the Centre for Genomic Research (University of Liverpool).

### Analysis of RNAseq read data

Illumina reads (ArrayExpress accession E-MTAB-4814) were quality trimmed and mapped to the Cyanorak curated *Synechococcus* sp. BL107 genome (www.sb-roscoff.fr/cyanorak/) using CLC Genomics Workbench 8.0.1 (Qiagen). Briefly, reads were quality trimmed before global read mapping to the *Synechococcus* sp. BL107 genome with the following altered mapping stringencies: mismatch penalty 3, deletion cost 3, length fraction 0.9, similarity fraction 0.9. Counting of both paired and singlet reads that mapped to protein coding regions only gave total reads per gene. The reads mapping over a gene boundary were counted toward the total expression value for the gene they best mapped to. Gene counts for each sample were then normalized and compared to the control (RNA extracted from the material that had not been filtered or sorted) using the DESeq2 package in R (Love et al., 2014). The four copies of the *psbA* gene were excluded from the analysis due to their high degree of sequence similarity (95%) at the nucleotide level. Combined with a short transcript read length and the nature by which reads are assigned when they map equally well to multiple regions, it is not possible to accurately distinguish changes in transcript ratios between the four copies (Garczarek et al., 2008).

## Results

### SWiFT: an effective filtration rig for the rapid recovery of marine picoplankton

SWiFT (Figure 1) is a unique three-stage water filtration unit designed to concentrate and recover picoplankton in liquid from large volumes of seawater in a short space of time. Seawater is propelled through the system via a peristaltic pump, undergoing an initial filtration to remove large biomass >35 μm, a second stage which removes air bubbles from the system and finally a third stage in which cells (>0.2 μm) are captured via a parallel 0.22 μm diameter pore size hollow fiber filter (CTHV400, Celltrap™, MEMTEQ Ventures Ltd. UK).

Initial cell sorting trials with *Synechococcus* sp. BL107 revealed a minimum of 1.5 × 10^7^ cells were required for sufficient RNA recovery to meet minimum total RNA yields for the ScriptSeq v2 (Epicentre) library preparation without the need for amplification. Based on the lowest measurements for open ocean *Synechococcus* cell numbers 1 × 10^3^ cells ml^−1^ (Partensky et al., 1999; Zubkov et al., 2007; Zwirglmaier et al., 2008) and an approximation of 93% cell recovery (Table 1) we estimated that 200 l of seawater would need to be processed within a 20 min time frame, inclusive of water transfer times and cell freezing.

**Table 1:** **SWiFT rig pre-filtration cell retention trials**.

**Pre-filter specification**				***Synechococcus*** **sp. BL107 cell counts (cells ml^−1^)**			
**Name/brand**	**Filter type**	**Pore size (μm)**	**Sample No**.	Pre Filtration^**^	**CT400 Recovered Cells Post Filtration**	**% Recovery**	**% Average**
Nuclepore	membrane	5	1	182,557	178,555	98	
			2	186,240	172,572	93	95
Supagard	spun bonded	20	1	350,782	221,167	63	
			2	336,710	217,830	65	64
Supaspun II	spun bonded	20	1	153,292	94,942	62	
			2	168,942	73,807	44	53
Supapleat II	pleat (PP^*^)	20	1	111,187	75,870	68	
			2	113,995	73,807	65	66
Supapleat II	pleat (PP)	35	1	90,245	86,553	96	
			2	99,242	88,433	89	93

* *Polypropelene*.

** *cell counts based on an avaerage of three flow counts. Four graded depth filters were tested on the SWiFT rig to assess cell retention properties for comparison with flat membrane filtration (blue)*.

While transcripts turning over at the timescale of a few minutes are not captured by most current sampling devices i.e., except for autosamplers (Jacquet et al., 1998), a 20 min window was deemed effective for capturing the majority of transcripts with half-lives in the midrange based on a previous study of the RNA half-life from *Prochlorococcus* (Steglich et al., 2010). Flat membrane filters commonly used for bacterial community recovery were not able to handle the biomass load or speed of filtration. A significant advantage of the hollow fiber filter is that concentrated cells can be efficiently recovered in ~20 s in liquid, using a syringe, whereas recovering the same biomass from a large flat filter would be considerably slower with a lower efficiency.

Celltraps that consist of parallel hollow fiber filter tubing designed for dead end filtration (Figure 1C) have been routinely used for cell recovery in liquid (Gómez-Pereira et al., 2013). While the CT40 model is still restrictive with a 2–20 l seawater capacity, the new high volume CT400, with an estimated equivalent surface area of 100,000 mm^2^ is now able to meet both volume and time restrictions processing up to 300 l within 20 min when used in conjunction with a 620SN/R pump (Watson Marlow) and a 15.9 mm bore silicone tubing.

We used a pre-filtration step to remove particles larger than 35 μm through a large capacity cartridge pleat filter (Amazon Filters Ltd). Industrial pleat filters are graded to prevent passage of particles above a target size range. However, smaller particle retention rates were unknown. Both pleat and flat membrane filtration trials were carried out using cultured *Synechococcus s*p. BL107 cells to assess recovery rates. Data showed that the Supapleat II (35 μm pore graded) pleat filter gave comparable cell recovery rates to a Nuclepore flat membrane filtration system (Table 1).

### Changes to the *Synechococcus* transcription profile caused by filtration were limited and well-defined

A trial protocol was designed to isolate and assess the impact of a 20-min HVCT cell concentration, combined with a frozen sample transit period, followed by the cumulative impact of FACS on the transcriptome of *Synechococcus* sp. BL107. The RNA yield following extraction was low (< 35 ng), which ruled out the possibility to deplete the rRNA pool by subtractive hybridisation, with the risk of biasing the resultant mRNA pool (Giannoukos et al., 2012). Total RNA was therefore sequenced with no prior amplification on the Illumina Hi-Seq platform (Supplementary Table 1).

**Figure 3:**
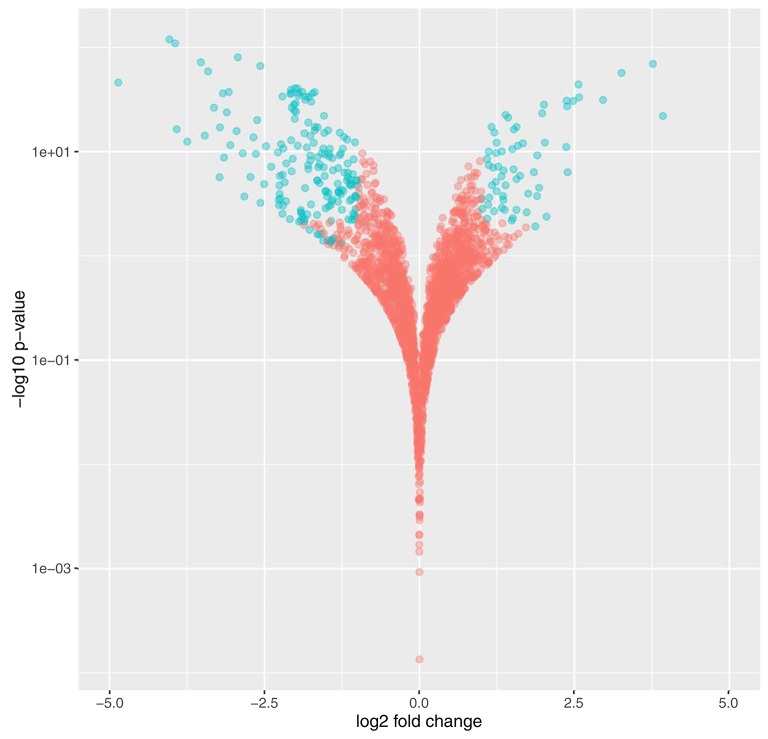
**Volcano plot demonstrating significant changes to the *Synechococcus* sp. BL107 transcriptome post SWiFT filtration**. Genes demonstrating < 0.05 *q*-value and >2-fold change in gene expression are highlighted in blue.

Maximum and average total read counts for each of the conditions demonstrated comprehensive coverage of the *Synechococcus* sp. BL107 transcriptome with 6% of genes (150) on average across all conditions reporting no counts (Table 2). Comparison of the SWiFT filtered with the unfiltered *Synechococcus* sp. BL107 transcriptome showed 9% (226) of protein encoding genes demonstrated a >2-fold change (*q* < 0.05) in their expression levels (Figure 3). This is a relatively limited gene set that is being affected, indeed, when compared to nutrient starvation (e.g., iron stress in *Synechococcus* sp. BL107 causes the differential expression of 15% of genes (Millard, Eriksson and Scanlan unpublished data).

**Table 2:** **RNASeq read counts**.

	**Control**	**SWiFT Filtration**	**SWiFT Filtration + Single Sort**	**SWiFT Filtration + Double Sort**
TOTAL READ COUNTS^**^				
Maximum^*^	16,377	64,695	15,362	16,252
Mean	69	335	62	59
Median	18	102	17	16
**TOTAL GENE COUNT**				
Read count = 0	150	101	177	173

* *Maximum counts reported for a single gene*.

** *Average paired and singlet read counts across protein coding regions. Total read counts reported using the CLC bio count strategy as described in Materials and Methods*.

Close to half (48%, 107) of the genes significantly altered had known annotations within the Kyoto Encyclopaedia of Genes and Genomes (KEGG) allowing further categorisation into 15 broad functional groups (Table 3). Five subgroups were found to contain a significant enrichment of genes known to be associated with a particular pathway or functional cluster (Fisher's exact test, Table 4). These groups were: photosynthetic antenna, photosynthetic core, phycobilin metabolism, chaperones, and nitrogen metabolism associated genes. All of the above groups demonstrated a decrease in expression with the exception of the photosynthetic core associated ferredoxin (*petF*), circadian regulators *rpaA*, and primary sigmaF2, and all of the chaperone proteins.

**Table 3:** **Functional groups defining the effect of SWiFT filtration on the transcriptome profile of *Synechococcus* sp. BL107**.

**Gene ID**	**GI number**	**Gene or Predicted function**	**Log2 (Fold Change)**	**lfcSE**
**1: TRANSLATION**				
BL107_08616	116,064,447	RP-S19; small subunit ribosomal protein S19	−1.11	0.26
BL107_07804	116,065,662	rluD; 23S rRNA pseudouridine synthase	1.35	0.36
BL107_17155	116,066,800	PDF; peptide deformylase	1.13	0.31
**2: TRANSCRIPTION**				
BL107_15780	116,066,525	yhbH; putative sigma-54 modulation protein	−1.17	0.26
BL107_06794	116,065,460	rpoD4; type II alternative RNA polymerase sigma factor (sigma 70)	−1.17	0.16
**3: REPLICATION, RECOMBINATION AND REPAIR**				
BL107_09206	116,064,565	mutM; formamidopyrimidine-DNA glycosylase	−1.51	0.38
BL107_05069	116,065,115	DPO3D2; DNA polymerase III subunit delta'	−1.30	0.33
BL107_12076	116,065,047	rmuC; DNA recombination protein RmuC	3.26	0.20
BL107_11686	116,064,969	recD; exodeoxyribonuclease V alpha subunit	1.09	0.39
BL107_06249	116,065,351	uvrB; exonuclease ABC subunit B	1.00	0.28
**8: SIGNAL TRANSDUCTION MECHANISMS**				
BL107_13595	116,066,088	typA; GTP-binding protein	1.33	0.21
**8.1: CIRCADIAN CLOCK SIGNALING**				
BL107_05064	116,065,114	rpaA; two-component system, OmpR family, response regulator RpaA	−1.23	0.18
BL107_06099	116,065,321	SIGF2; RNA polymerase non-essential primary-like sigma factor	2.96	0.25
BL107_06029	116,065,307	rpaB; two-component system, OmpR family, response regulator RpaB	1.39	0.32
**10: CELL MOTILITY**				
BL107_07554	116,065,612	pilT; twitching motility protein PilT	−1.01	0.24
**14: POST TRANSLATIONAL MODIFICATION, PROTEIN TURNOVER, CHAPERONES, DETOXIFICATION**				
**14.1: PROTEIN TURNOVER**				
BL107_08139	116,065,729	Peptidase M41	1.12	0.20
BL107_14420	116,066,253	clpB; ATP-dependent Clp protease ATP-binding subunit ClpB	1.12	0.17
**14.2: CHAPERONES**				
BL107_06544	116,065,410	Heat shock protein DnaJ	1.59	0.23
BL107_06549	116,065,411	Putative heat shock protein GrpE	1.24	0.31
BL107_12790	116,065,927	Heat shock protein 90	1.24	0.17
BL107_16590	116,066,687	Chaperonin GroEL	1.39	0.14
BL107_16595	116,066,688	co-chaperonin GroES	1.23	0.28
BL107_09846	116,064,693	Chaperonin Cpn60/TCP-1	1.21	0.15
BL107_06744	116,065,450	Molecular chaperone DnaK	1.16	0.13
**14.3: DETOXIFICATION**				
BL107_06109	116,065,323	Glutathione peroxidase	1.01	0.32
BL107_13155	116,066,000	Thioredoxin peroxidase	1.09	0.18
**15: ENERGY PRODUCTION AND CONVERSION**				
**15.1: PHOTOSYNTHESIS: ANTENNA PROTEINS**				
BL107_08876	116,064,499	cpcA; phycocyanin alpha chain	−2.21	0.18
BL107_08996	116,064,523	Possible phycobilisome linker polypeptide	−2.08	0.17
BL107_08946	116,064,513	C-phycoerythrin class II gamma chain	−2.02	0.18
BL107_08906	116,064,505	cpeB; phycoerythrin beta chain	−2.01	0.15
BL107_08881	116,064,500	cpcB; phycocyanin beta chain	−2.00	0.18
BL107_08991	116,064,522	cpeS; phycoerythrin-associated linker protein	−1.99	0.19
BL107_09001	116,064,524	cpeD; phycoerythrin-associated linker protein	−1.95	0.16
BL107_09006	116,064,525	cpeC; phycoerythrin-associated linker protein	−1.84	0.16
BL107_13065	116,065,982	apcD; allophycocyanin-B	−1.79	0.20
BL107_17205	116,066,810	cpcG; phycobilisome rod-core linker protein	−1.78	0.15
BL107_16740	116,066,717	apcE; phycobilisome core-membrane linker protein	−1.75	0.15
BL107_08956	116,064,515	C-phycoerythrin class II beta chain	−1.73	0.14
BL107_09071	116,064,538	cpcC; phycocyanin-associated rod linker protein	−1.69	0.20
BL107_08951	116,064,514	cpeA; phycoerythrin alpha chain	−1.69	0.13
BL107_08986	116,064,521	cpeT; CpeT protein	−1.68	0.46
BL107_16755	116,066,720	apcC; phycobilisome core linker protein	−1.65	0.36
BL107_09016	116,064,527	cpcG; phycobilisome rod-core linker protein	−1.61	0.29
BL107_13735	116,066,116	apcF; phycobilisome core component	−1.55	0.29
BL107_08911	116,064,506	cpeA; phycoerythrin alpha chain	−1.53	0.19
BL107_16750	116,066,719	apcB; allophycocyanin beta subunit	−1.46	0.24
BL107_08941	116,064,512	cpeU; bilin biosynthesis protein	−1.32	0.38
BL107_16745	116,066,718	apcA; allophycocyanin alpha subunit	−1.13	0.26
**15.2 PHOTOSYNTHESIS: CORE PROTEINS**				
BL107_10506	116,064,733	psaM; photosystem I subunit XII	−3.23	0.68
BL107_12730	116,065,915	psaK; photosystem I subunit X	−2.93	0.15
BL107_09957	116,064,350	psaJ; photosystem I subunit IX	−2.73	0.57
BL107_08771	116,064,478	psaD; photosystem I subunit II	−2.57	0.15
BL107_05514	116,065,204	psbL; photosystem II PsbL protein	−2.39	0.44
BL107_08424	116,065,786	psaL; photosystem I subunit XI	−2.06	0.16
BL107_05504	116,065,202	psbE; photosystem II cytochrome b559 subunit alpha	−1.96	0.28
BL107_14915	116,066,352	PTOX; plastoquinol terminal oxidase	−1.94	0.15
BL107_09962	116,064,351	psaF; photosystem I subunit III	−1.89	0.15
BL107_05509	116,065,203	psbF; photosystem II cytochrome b559 subunit beta	−1.75	0.52
BL107_08429	116,065,787	psaI; photosystem I subunit VIII	−1.65	0.56
BL107_08409	116,065,783	psaA; photosystem I P700 chlorophyll a apoprotein A1	−1.64	0.19
BL107_04827	116,064,343	psbU; photosystem II PsbU protein	−1.63	0.30
BL107_16480	116,066,665	psbV; photosystem II cytochrome c550	−1.55	0.23
BL107_09906	116,064,705	petA; apocytochrome f	−1.47	0.18
BL107_15730	116,066,515	psbD; photosystem II P680 reaction center D2 protein	−1.46	0.23
BL107_05519	116,065,205	psbJ; photosystem II PsbJ protein	−1.42	0.41
BL107_09211	116,064,566	psaE; photosystem I subunit IV	−1.32	0.30
BL107_09181	116,064,560	petD; cytochrome b6-f complex subunit 4	−1.27	0.20
BL107_09176	116,064,559	petB; cytochrome b6	−1.25	0.23
BL107_05834	116,065,268	psaC; photosystem I subunit VII	−1.21	0.24
BL107_09101	116,064,544	psbB; photosystem II CP47 chlorophyll apoprotein	−1.04	0.14
BL107_06219	116,065,345	psbZ; photosystem II PsbZ protein	−1.03	0.27
BL107_10302	116,06419	petF; Ferredoxin (2Fe-2S)	1.09	0.21
BL107_16490	116,066,667	petF; Ferredoxin (2Fe-2S)	1.22	0.22
**16: CARBOHYDRATE TRANSPORT AND METABOLISM**				
**16.1: CARBOHYDRATE METABOLISM**				
BL107_07599	116,065,621	pgk; phosphoglycerate kinase	1.57	0.34
BL107_15060	116,066,381	fba; fructose-bisphosphate aldolase	1.50	0.23
**17: AMINO ACID TRANSPORT AND METABOLISM**				
**17.1: GENERAL AMINO ACID TRANSPORT**				
BL107_14770	116,066,323	Extracellular solute-binding protein, family 3	1.11	0.33
**18: NUCLEOTIDE TRANSPORT AND METABOLISM**				
**18.1: PYRIMIDINE METABOLISM**				
BL107_08856	116,064,495	panC-cmk; pantoate ligase / cytidylate kinase	1.20	0.39
**19: CO-ENZYME / CO-FACTOR TRANSPORT AND METABOLISM**				
**19.1: FOLATE METABOLISM**				
BL107_10476	116,064,727	GCH1; GTP cyclohydrolase I	1.52	0.18
**19.2: COBALAMIN, HEME, PHYCOBILIN AND PORPHYRIN METABOLISM**				
BL107_05669	116,065,235	HMOX; heme oxygenase (biliverdin-producing)	−2.07	0.16
BL107_08791	116,064,482	CPOX; coproporphyrinogen III oxidase	−1.70	0.24
BL107_10526	116,064,737	chlL; light-independent protochlorophyllide reductase iron-sulfur ATP-binding protein	−1.57	0.25
BL107_10521	116,064,736	Protochlorophyllide reductase	−1.54	0.16
BL107_08891	116,064,502	pebA; 15,16-dihydrobiliverdin:ferredoxin oxidoreductase	−1.23	0.31
BL107_13240	116,066,017	Magnesium-protoporphyrin IX monomethyl ester (oxidative) cyclase	−1.22	0.16
BL107_14385	116,066,246	hemE; uroporphyrinogen decarboxylase	−1.15	0.24
**20: LIPID / MEMBRANE TRANSPORT AND METABOLISM**				
BL107_07019	116,065,505	gpgS; glucosyl-3-phosphoglycerate synthase	−2.06	0.19
BL107_06684	116,065,438	Sucrose-phosphate synthase	−1.39	0.27
BL107_12250	116,065,819	ACSS; acetyl-CoA synthetase	−1.10	0.30
BL107_11081	116,064,848	UDP-N-acetylglucosamine 1-carboxyvinyltransferase	1.19	0.22
BL107_14110	116,066,191	crtW; beta-carotene ketolase (CrtW type)	1.85	0.37
**21: INORGANIC ION TRANSPORT AND METABOLISM**				
**21.2 NITROGEN METABOLISM**				
BL107_06834	116,065,468	nirA; ferredoxin-nitrite reductase	−3.94	0.18
BL107_06819	116,065,465	cynS; cyanate lyase	−3.22	0.37
BL107_06989	116,065,499	urtA; urea transport system substrate-binding protein	−3.08	0.24
BL107_07964	116,065,694	NtcA; transcriptional regulator	−2.28	0.36
BL107_05259	116,065,153	amt; ammonium transporter, Amt family	−2.02	0.19
BL107_06904	116,065,482	nrtP; MFS transporter, NNP family, nitrate/nitrite transporter	−1.97	0.15
BL107_07009	116,065,503	urtE; urea transport system ATP-binding protein	−1.86	0.23
BL107_06954	116,065,492	ureC; urease subunit alpha	−1.82	0.27
BL107_16790	116,066,727	glnB; nitrogen regulatory protein P-II 1	−1.76	0.23
BL107_06964	116,065,494	ureA; urease subunit gamma	−1.51	0.47
BL107_06969	116,065,495	ureD; urease accessory protein	−1.47	0.45
BL107_06999	116,065,501	urtC; urea transport system permease protein	−1.26	0.31
**24: GENERAL FUNCTION PREDICTION**				
BL107_17417	116,066,849	Conserved hypothetical gene in phycobilisome gene region	−2.62	0.28
BL107_08174	116,065,736	UMPS; uridine monophosphate synthetase	−1.69	0.20
BL107_16080	116,066,585	Putative arylsulfatase regulatory protein	−1.02	0.28
BL107_12101	116,065,052	Superfamily I DNA/RNA helicase	2.57	0.18
BL107_14195	116,066,208	Putative oxidoreductase	1.90	0.51
**tRNA**				
BL107_t08537	–		−2.27	0.64
BL107_t17419	–		−2.27	0.68
BL107_t09927	–		−2.15	0.61
BL107_t10374	–		−1.91	0.61
BL107_t12185	–		−1.89	0.66
BL107_t17423	–		−1.86	0.69

*Samples which underwent SWiFT filtration were compared with the control samples where no processing had taken place. Genes with Prochlorococcus sp. MED4 counterparts known to oscillate as part of the daily circadian rhythm are shaded in gray (Zinser et al., 2009)*.

**Table 4:** **Functional group enrichment**.

Cyanobase Categories/Subcategories^*^	**Gene counts (Enriched/Total)**	Enrichment (*P*-value)^**^
1: Translation	3/79	0.1531
2: Transcription	3/50	0.4362
3: Replication, recombination and repair	5/92	0.3457
8: Signal transduction mechanisms	1/28	0.727
8.1: Circadian clock signaling	3/9	0.0695
10: Cell motility	1/10	0.6075
14.1: Protein turnover	2/18	0.675
14.2: Chaperones	7/16	0.0017
14.3: Detoxification	2/7	0.1671
15.1: Photosynthesis: Antenna Proteins	22/26	2.0527E−12
15.2 Photosynthesis: Core Proteins	25/63	7.6303E−09
16.1: Carbohydrate metabolism	2/54	0.3188
17.1: General amino acid transport	1/4	0.3459
18.1: Pyrimidine metabolism	1/34	0.3589
19.1: Folate metabolism	1/13	1.0000
19.2: Cobalamin, heme, phycobilin, and porphyrin metabolism	7/44	0.0003
20: Lipid transport and metabolism	5/53	0.8083
21.1: Porphyrin/Chlorophyll Metabolism	5/28	0.1849
21.2 Nitrogen Metabolism	12/22	5.8977E−06

* *Functional Categories from Cyanobase for Synechococcus sp. WH7803 were used with the addition of subcategories where applicable*.

** *Calculations based on two tailed Fisher's exact test. Fisher's exact test was applied to each of the functional groups to assess if a significant enrichment of genes was present*.

Changes to the gene expression profile appear well-defined when put in the context of the filtration process. Transfer of *Synechococcus* sp. BL107 through the SWiFT rig involves pumping the water through the apparatus at pressure. Water pressure entering the HVCT increases gradually (up to 25 psi) as the bacterial load builds up into a film on the cell trap restricting water flow. This would account for the increase in chaperone and heat shock associated proteins observed in the profile, corresponding to the bacterial response to moderate increases in hydrostatic pressure (Welch et al., 1993). Cells traveling through the filtration rig enter the cell trap filaments, which effectively place them in darkness for a period of up to 20 min depending on the time they spend within the filament core. The movement of cells into the dark filament cores has by far the largest impact on the transcriptome of *Synechococcus* sp. BL107. Indicators that this transition could be occurring can be seen in the decrease in expression of *rpaA* and increase in *rpaB* (Table 3). These two key response regulators are known to function cooperatively as part of the circadian kaiC-associated output pathway (Hanaoka et al., 2012; Markson et al., 2013) and have been shown to demonstrate a similar day to night transcriptome profile in the closely related cyanobacterium *Prochlorococcus* sp. MED4 (Zinser et al., 2009).

While no change was observed in the *kaiABC* core circadian oscillating genes, the transition to dark is strongly reflected in the changes in the rest of the transcriptome profile. Most notably down-regulation of the photosynthetic antenna and core photosystem genes occurs in conjunction with the down-regulation of the nitrogen transport and metabolism pathways, all common features of the switch from day to night (Waldbauer et al., 2012). Notable absences from the profile include changes to genes associated with glycogen metabolism, pentose phosphate pathway and the Calvin cycle. These genes together play important roles in orchestrating the cells' shift to the major night time activity of respiratory metabolism (Diamond et al., 2015). Failure to detect all common genes involved in the day to night transition is perhaps to be expected as typical circadian gene levels oscillate over a 24-h period whereas the BL107 cells only experience a dark period up to 20 min during SWiFT filtration.

### Flow sorting has minimal effect on the *Synechococcus* sp. BL107 transcriptome profile

Following both a single and double round of FACS only 5 and 6 genes, respectively changed their expression levels (q < 0.05, fold change >2) when compared with the post SWiFT filtration transcriptome profile (Table 5) The fact that changes to the transcription profile are extremely limited provides good evidence that the rapid thaw and cell sorting periods effectively preserve the transcript profile post SWiFT filtration at the point of snap freezing.

**Table 5:** **Genes demonstrating a change in expression post SWiFT filtration following both a single and double round of flow cytometric sorting**.

**Gene ID**	**GI number**	**Gene or Predicted function**	**Log2 (Fold change)**	**lfcSE**
**SWiFT FILTRATION VS. SWiFT FILTRATION** + **SINGLE CELL SORT**				
BL107_05779	116,065,257	Lipopolysaccharide biosynthesis protein-like	−1.35	0.39
BL107_13430	116,066,055	Hypothetical protein	1.37	0.22
BL107_09972	116,064,353	Putative high light inducible protein	1.32	0.37
BL107_15205	116,066,410	Hypothetical protein	1.06	0.25
BL107_07309	1160,65,563	Hypothetical protein	1.01	0.20
**SWiFT FILTRATION VS. SWiFT FILTRATION** + **DOUBLE CELL SORT**				
BL107_05889	116,065,279	Possible oxidoreductase, GFO/Idh/MocA family	−1.06	0.28
BL107_13430	116,066,055	Hypothetical protein	1.59	0.28
BL107_15730	116,066,515	Photosystem II reaction center protein PsbD/D2	1.25	0.24
BL107_15205	116,066,410	Hypothetical protein	1.18	0.28
BL107_06924	116,065,486	Hypothetical protein	1.02	0.30
BL107_05839	116,065,269	Acyl-carrier protein	1.00	0.26

**Figure 4:**
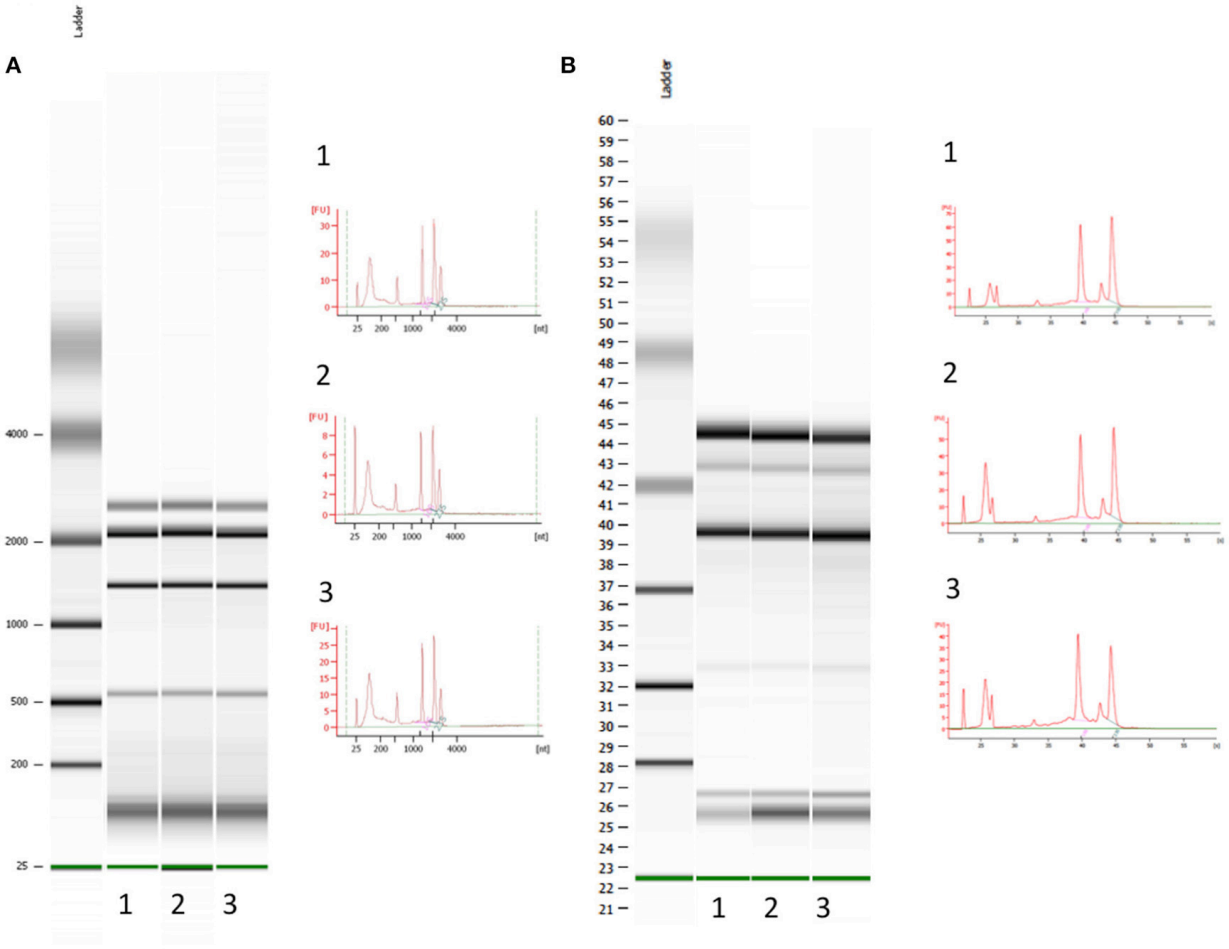
**Bioanalyzer electrophoretogram output for prokaryotic total RNA fragment analysis. (A)** Control samples: cells which underwent immediate RNA extraction after removal from the culture; samples run on a nano 6000 chipset **(B)** Samples post SWiFT filtration, 16 weeks frozen at −80°C and a single round of flow sorting; samples run on a pico 6000 chipset.

To our knowledge, this is the first study in which sorted cells are frozen immediately after drop deflection from the main sort stream as a result of a custom designed cooling chamber (Figure 2). This effectively reduces total cell thaw time by up to two-thirds compared with a standard 20-min workflow for a 1 ml sample from thaw to final sort collection. Prior to FACS, cells were stored at −80°C for 16 weeks to replicate sample transit times during long cruise sampling transects. While RNA yield comparisons between the control and sorted samples are complicated by the fact that the cells were treated differently prior to RNA extraction, Bioanalyzer analysis demonstrates RNA integrity remains constant following −80°C storage for 16-weeks (Figure 4). The observed limited changes in the transcript profile also suggest RNA degradation during extended periods of freezing is limited, at least in the case of *Synechococcus* sp. BL107.

## Discussion

### Meeting the need for an effective affordable filtration rig

The ability to sample the aquatic microbial community has been in place for over 30 years thanks to remotely operated bottle samplers mounted on observational instruments (James Baker, 1981). These devices monitor **c**onductivity (salinity), **t**emperature and underwater pressure (**d**epth) (CTD) in real time allowing researchers to be selective with respect to the position in the water column sampled. Traditionally, water samples are manually taken from the CTD mounted Niskin bottles or pumped directly onboard the ship. Pico and micro-planktonic community cell capture then occurs via vacuum or gravity filtration onto flat membrane filters or concentrated with tangential flow filtration (Schmidt et al., 1991; Ganesh et al., 2014).

Technological advancements in recent years have moved water filtration apparatus from manned on-ship rigs to automated remote moorings able to remain at sea for months at a time (Scholin et al., 2009; Harvey et al., 2012), allowing access to water bodies otherwise inaccessible (Winslow et al., 2014). Filtration devices are even mounted on long-range autonomous underwater vehicles (AUV) able to descend the water column down to 4000 m (http://www.mbari.org/esp-technical-information/). For cruise transect sampling, robotic filtration devices are now being mounted directly onto the on-ship CTD, negating transit times required to bring deep water to the surface (Lauro et al., 2014).

While impressive in their ability to remotely sample, these advanced deployments come with two significant drawbacks. The first involves scale and recoverability. Most on-board filtration units use either flat membrane or sterivex™ filters (Millipore) with a limited capacity and poor recovery of cells for the purposes of cell sorting. These constraints effectively restrict downstream genetic studies to whole community analysis. The second drawback, which applies to the wider research community, is the build costs and expertise required for operation. At the budget end of the spectrum, development and build costs of robotic samplers is estimated around $200,000 (Lauro et al., 2014) and requires a skilled technician to maintain and operate the equipment. In this study, we have developed an alternative low cost (total build $5000), high-speed cell sampler (Figure 1) that needs no specialist skills to build, operate, and maintain. We have been able to demonstrate that SWiFT cell capture not only provides an effective planktonic cell recovery system but for the first time that post filtration cell sorting does little to either degrade or change the RNA transcript profile of a marine cyanobacterium. Application of this complete ocean sampling to sequence protocol for different phytoplankton groups could become increasingly attractive in the future as new multi-laser cytometers are now able to differentiate natural populations of phytoplankton at the class level (Thompson and van den Engh, 2016).

### The effects of SWiFT cell capture and how they can be accommodated within an environmental sampling context

The effect of filtration on the transcription profile (Table 3) is well-defined when placed in context with the movement of cells into the dark filament cores of the HVCT concentrating device. The down-regulation of photosynthesis genes and up-regulation of genes involved in respiration is congruent with the known circadian cell programmed shift from day to night and warrants important considerations when sampling in an environmental context. CTD Niskin collection bottles are opaque effectively placing any sample collected by them into the dark. The period of time any given sample spends in the bottle is then dependent on both the depth sampled and the number of sample depths completed within the column profile. CTD transit times can take anywhere between 5 and 40 min from the point of sampling to on-deck retrieval. Cell trap filtration devices are also not alone in their reduced opacity, with several of the membrane filter holders on the market completely encapsulating flat membrane filters within a stainless steel or opaque plastic holder. Given the findings of this study, it would perhaps be unwise to perform a diel cycle sampling program using SWiFT cell capture. However, because the impact to the transcriptome is both limited (with 9% of genes affected for our model strain *Synechococcus* sp. BL107) and well-defined (light-dark transition), this can be accounted for when assessing the transcriptome of environmental samples.

The ability to account for sampling impacts on cellular processes is paramount if we are to derive meaningful data within an environmental context. Taking into account the effects of sampling lends confidence to our ability to detect both temporal and spatial changes in a targeted community profile. Moving toward the next stage of testing with open ocean trials, this genus targeted approach aims to identify clade-specific gene sets, with the opportunity to uncover those actively being expressed across different geographic provinces. This method will help to explore the role that specific gene sets play in defining the ecological distinctness of these lineages, giving new insights into how *Synechococcus* populates the environment.

## Author contributions

FP and DS designed and conceived the project and critically evaluated practical work at all stages. FP conducted all practical work and data analysis. AM contributed to analysis and Interpretation of data. MO contributed to conceptual design and implementation of filtration apparatus. SD contributed to conceptual design of the FACS custom collection chamber. SM contributed to the design of pipeline trials. MZ contributed to field sampling application. FP and DS wrote the manuscript. All authors critically evaluated the manuscript.

## Funding

The research performed in this manuscript was funded by National Environment Research Council grant NE/I00985X/1.

### Conflict of interest statement

The authors declare that the research was conducted in the absence of any commercial or financial relationships that could be construed as a potential conflict of interest.

## Abbreviations

HVCT: High Volume Celltrap
SWiFT: High Speed Water Filtration
NGS: next generation sequencing
CTD, conductivity (salinity): temperature and underwater pressure (depth)
ASW: artificial seawater.
