# Supplementary material for: A Sample-to-Sequence Protocol for Genus Targeted Transcriptomic Profiling: Application to Marine Synechococcus
Source: Front Microbiol. 2016 Oct 14;7:1592. doi: 10.3389/fmicb.2016.01592 (PMC5063861; doi:10.3389/fmicb.2016.01592)
Supplement: Supplementary file 1 [file Table1.docx]

**Supplementary Information:**

**Supplementary Table legends:**

**Table 1: RNASeq read abundances:** 2 x 100 bp reads recovered from HiSeq2000 runs (V3 chemistry).

| Treatment | Paired Reads | | Singlet Reads | Total Reads | % GC |
| --- | --- | --- | --- | --- | --- |
|  | R1 | R2 | R0 |  |  |
|  |  |  |  |  |  |
| Control | 17,005,400 | 17,005,400 | 287,909 | 34,298,709 | 54 |
| Control | 16,653,484 | 16,653,484 | 319,843 | 33,626,811 | 54 |
| Control | 16,721,865 | 16,721,865 | 259,820 | 33,703,550 | 54 |
| SWiFT Filtration | 94,390,124 | 94,390,124 | 2,662,722 | 191,442,970 | 54 |
| SWiFT Filtration | 91,051,972 | 91,051,972 | 2,235,929 | 184,339,873 | 54 |
| SWiFT Filtration | 81,713,753 | 81,713,753 | 1,631,218 | 165,538,724 | 54 |
| SWiFT Filtration + Single Sort | 17,116,648 | 17,116,648 | 305,211 | 34,538,507 | 54 |
| SWiFT Filtration + Single Sort | 16,719,940 | 16,719,940 | 294,347 | 33,734,227 | 54 |
| SWiFT Filtration + Single Sort | 16,721,865 | 16,721,865 | 259,820 | 33,703,550 | 54 |
| SWiFT Filtration + Double Sort | 17,686,844 | 17,686,844 | 304,806 | 35,678,494 | 54 |
| SWiFT Filtration + Double Sort | 15,371,912 | 15,371,912 | 245,098 | 30,988,922 | 54 |
